# Correction: Koush et al. Chitosan-Stabilized Lipid Vesicles with Indomethacin for Modified Release with Prolonged Analgesic Effect: Biocompatibility, Pharmacokinetics and Organ Protection Efficacy. *Pharmaceutics* 2025, *17*, 523

**DOI:** 10.3390/pharmaceutics17081059

**Published:** 2025-08-15

**Authors:** Angy Abu Koush, Eliza Gratiela Popa, Beatrice Rozalina Buca, Cosmin Gabriel Tartau, Iulian Stoleriu, Ana-Maria Raluca Pauna, Liliana Lacramioara Pavel, Paula Alina Fotache, Liliana Mititelu Tartau

**Affiliations:** 1 Department of Pharmacology, Faculty of Medicine, ‘Grigore T. Popa’ University of Medicine and Pharmacy, 700115 Iasi, Romania; maierean_angy@yahoo.com (A.A.K.); beatrice-rozalina.buca@umfiasi.ro (B.R.B.); lylytartau@yahoo.com (L.M.T.); 2 Department of Pharmaceutical Technology, Faculty of Pharmacy, ‘Grigore T. Popa’ University of Medicine and Pharmacy, 700115 Iasi, Romania; 3 Department of Histology, Faculty of Medicine, ‘Grigore T. Popa’ University of Medicine and Pharmacy, 700115 Iasi, Romania; cosmin.tartau@gmail.com; 4 Faculty of Mathematics, ‘Alexandru Ioan Cuza’ University, 700506 Iasi, Romania; iulian.stoleriu@uaic.ro; 5 Department of Anatomy, Faculty of Medicine, ‘Grigore T. Popa’ University of Medicine and Pharmacy, 700115 Iasi, Romania; paunaanamariaraluca@gmail.com; 6 Medical Department, Faculty of Medicine and Pharmacy, ‘Dunarea de Jos’ University, 800010 Galati, Romania; doctorpavel2012@yahoo.com; 7 Department of Morphological and Functional Sciences, Faculty of Medicine and Pharmacy, ‘Dunarea de Jos’ University, 800010 Galati, Romania; fotache.paula@yahoo.com

## Error in Figure

In the original publication [1], there were mistakes in Figures 1–3 as published. Accidental duplication of narrow image regions occurred during multiple rounds of graphic revisions and layout changes, resulting in partial overlaps at the outer edges. The corrected Figure 1, Figure 2 and Figure 3 appear below. The authors state that the scientific conclusions are unaffected. This correction was approved by the Academic Editor. The original publication has also been updated.

## Figures and Tables

**Figure 1:**
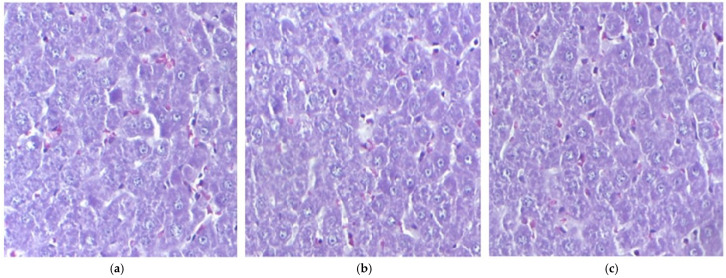
The histological liver structure in mice belonging to the following groups: Control (**a**), IND (**b**), IND-ves (**c**). Masson trichrome stain ×20, scale bar = 100 µm.

**Figure 2:**
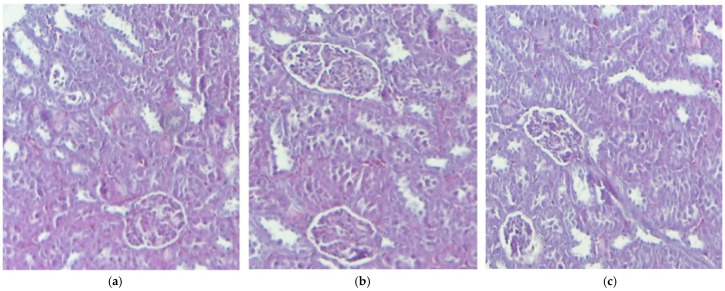
The histological kidney structure in mice belonging to the following groups: Control (**a**), IND (**b**), IND-ves (**c**). Masson trichrome stain ×20, scale bar = 100 µm.

**Figure 3:**
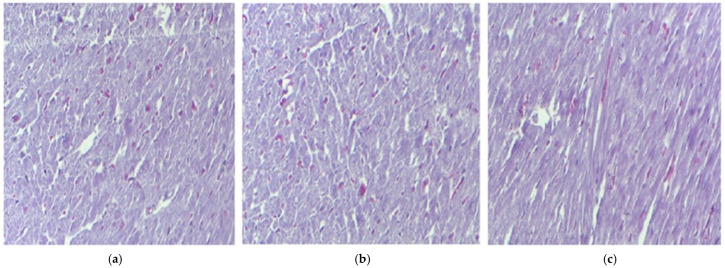
The histological myocardium structure in mice belonging to the following groups: Control (**a**), IND (**b**), IND-ves (**c**). Masson trichrome stain ×20, scale bar = 100 µm.

## References

[1] Koush A.A., Popa E.G., Buca B.R., Tartau C.G., Stoleriu I., Pauna A.-M.R., Pavel L.L., Fotache P.A., Tartau L.M. (2025). Chitosan-Stabilized Lipid Vesicles with Indomethacin for Modified Release with Prolonged Analgesic Effect: Biocompatibility, Pharmacokinetics and Organ Protection Efficacy. Pharmaceutics.

